# Defunctioning Stomas Result in Significantly More Short-Term Complications Following Low Anterior Resection for Rectal Cancer

**DOI:** 10.1007/s00268-018-4672-0

**Published:** 2018-05-17

**Authors:** Andrew Emmanuel, Ezzat Chohda, Christo Lapa, Andrew Miles, Amyn Haji, Joe Ellul

**Affiliations:** 1 Department of Colorectal Surgery, King’s College Hospital NHS Foundation Trust, London, UK; 2 Department of Colorectal Surgery, Hampshire Hospitals NHS Foundation Trust, Wessex, UK

## Abstract

**Background:**

Studies suggest that defunctioning stomas reduce the rate of anastomotic leakage and urgent reoperations after anterior resection. Although the magnitude of benefit appears to be limited, there has been a trend in recent years towards routinely creating defunctioning stomas. However, little is known about post-operative complication rates in patients with and without a defunctioning stoma. We compared overall short-term post-operative complications after low anterior resection in patients managed with a defunctioning stoma to those managed without a stoma.

**Methods:**

A retrospective cohort study of patients undergoing elective low anterior resection of the rectum for rectal cancer. The primary outcome was overall 90-day post-operative complications.

**Results:**

Two hundred and three patients met the inclusion criteria for low anterior resection. One hundred and forty (69%) had a primary defunctioning stoma created. 45% received neoadjuvant radiotherapy. Patients with a defunctioning stoma had significantly more complications (57.1 vs 34.9%, *p* = 0.003), were more likely to suffer multiple complications (17.9 vs 3.2%, *p* < 0.004) and had longer hospital stays (13.0 vs 6.9 days, *p* = 0.005) than those without a stoma. 19% experienced a stoma-related complication, 56% still had a stoma 1 year after their surgery, and 26% were left with a stoma at their last follow-up. Anastomotic leak rates were similar but there was a significantly higher reoperation rate among patients managed without a defunctioning stoma.

**Conclusion:**

Patients selected to have a defunctioning stoma had an absolute increase of 22% in overall post-operative complications compared to those managed without a stoma. These findings support the more selective use of defunctioning stomas.

**Study registration:**

Registered at www.researchregistry.com (UIN: researchregistry3412).

## Introduction

The routine use of defunctioning stomas during anterior resection has increased in recent years with several national audits and colorectal cancer registries demonstrating approximately 80% or more of patients undergoing anterior resection for rectal cancer are managed with defunctioning stomas [1–3]. Several studies suggest that patients who have a defunctioning stoma created during anterior resection have a lower risk of anastomotic leak, or a lower risk of requiring urgent reoperation, than patients who do not have a defunctioning stoma [4–7]. However, the quality of many of these studies is poor, the magnitude of this benefit is uncertain and it is not clear which patients may benefit most from a defunctioning stoma. Overall post-operative complications are rarely reported in these studies. There are very few studies specifically comparing the overall complications in patients managed with and without defunctioning stomas [8–10] and these generally involve small numbers of patients and report conflicting results. Despite the paucity of comparative studies, a number of case series suggest that there is significant short-term morbidity associated with defunctioning stomas [11–13].

Given that the majority of the large proportion of patients who are managed with a defunctioning stoma will not derive any benefit from it, it is important to understand if creating a stoma, which is a significant additional procedure in an already long and complex operation, is associated with an increase in overall complications. If patients with a defunctioning stoma have a clinically significant increase in overall complications compared to those who do not have a stoma, this may influence surgeons’ decisions regarding the benefit of defunctioning stomas during anterior resection and may lead to more selective use of stomas.

The aim of this study is to determine if patients who receive a defunctioning stoma during elective low anterior resection of the rectum for rectal cancer have a clinically significant increase in overall post-operative complications compared to patients who do not receive a defunctioning stoma.

## Materials and methods

### Study design and outcomes

This is a retrospective cohort study of consecutive patients with rectal cancer treated by elective low anterior resection of the rectum at a single hospital trust comprising two hospitals between 1st January 2009 and 31st December 2014. All procedures were performed or directly supervised by colorectal surgeons trained in TME. The procedures adhered to the principles of total mesorectal excision or tumour-specific modified mesorectal excision depending on the level of the tumour. In general, total mesorectal excision is performed for all middle and lower rectal cancers while modified tumour-specific mesorectal excision can be used for upper rectal cancers, with the mesorectum divided no less than 5 cm below the margin of the tumour [14]. Defunctioning stomas were created at the discretion of the operating surgeon. Perioperative management was based on an enhanced recovery after surgery protocol including essential principles as per ERAS society guidelines, including pre-operative counselling and education, medical optimisation, no overnight fasting, pre-operative carbohydrate loading drinks, standard management for premedication, analgesia and post-operative nausea and vomiting, venous thromboembolism prophylaxis, antibiotic prophylaxis, avoidance of nasogastric tubes and abdominal drains, early post-operative diet and early mobilisation [15]. Sample size calculation was performed based on detecting a clinically significant increase in the overall complication rate of 20% from 25 to 45% (RR 1.8) with a power 0.8 and alpha 0.05, assuming that 65% of patients would have a defunctioning stoma created at anterior resection. An increase in the complication rate of 20% was chosen as an increase in a magnitude significant enough to influence a surgeon’s decision regarding whether to routinely form a defunctioning stoma. The calculation yielded a necessary sample size of 197 patients (128 with a defunctioning stoma and 69 without).

The primary outcome was the overall post-operative complication rate within 90 days of surgery. Secondary outcomes included length of hospital stay, readmission, reoperation, multiple complications, mortality and stoma-related complications.

### Definition of low anterior resection

The Tripartite Consensus Conference on Definitions for Anorectal Physiology and Rectal Cancer definition of low anterior resection was used, which is a colorectal resection with anastomosis of the colon to extraperitoneal rectum (anastomosis below the peritoneal reflection) [16]. Although it is difficult to assign an exact level for the anterior peritoneal reflection in each individual, studies in living subjects place it at approximately 9 cm [17].

### Criteria to identify patients with a low anterior resection

Patients with tumours 12 cm or less from the anal verge were included as these patients are consistently likely to have a distal resection margin below the peritoneal reflection, which is consistent with the tumour height used in other studies of low anterior resection [18–20]. The tumour level was determined by the pre-operative MRI scan as MRI provides an objective, accurate and reproducible means of assessing rectal tumours [21] and documentation of the level of the tumour from the anal verge is now internationally accepted as a reporting standard for pre-operative MRI [22, 23]. If the tumour level was not documented pre-operatively then patients were included if histopathological analysis of the resection specimen clearly showed a distal resection margin below the anterior peritoneal reflection.

### Data collection

Data were collected by exhaustive review of patients’ clinical notes, operative record, post-operative follow-up visits and reports of radiological and histopathological findings. Data collected included patient demographics, comorbidities, ASA score, body mass index (BMI), pre-operative radiological staging, neoadjuvant therapy, surgical technique, date of stoma reversal, length of hospital stay, outcomes within 90 days of surgery including post-operative complications, mortality and reoperation, and histopathological staging and findings.

Short-term post-operative complications were defined as complications within 90 days of the primary operation and stratified according to the Clavien–Dindo classification [24]. Multiple complications were defined as ≥2 complications of any Clavien–Dindo grade experienced by the same patient. Ileus was defined as non-mechanical bowel obstruction requiring the passage of a nasogastric tube associated with high volume aspirates and bowel obstruction as radiological evidence of bowel obstruction with a transition point. High-output stoma was defined as production of ≥2000 ml of effluent per day.

### Data analysis

Comparisons were made between patients who received an initial defunctioning stoma at anterior resection and patients who did not receive a defunctioning stoma. Categorical data were analysed using the *Χ*^2^ test. Means of continuous data were analysed using the *t* test. Multiple logistic regression analysis was used to determine significant differences in characteristics between patients managed with and without a defunctioning stoma.

### Ethical approval and study registration

This study was approved by the National Research Ethics Service (REC reference 15/LO/1630). The study was registered with Research Registry (www.researchregistry.com).

## Results

Two hundred and fifty-nine patients underwent elective anterior resection of the rectum for rectal cancer. Two hundred and four patients met the study criteria for low anterior resection; one was excluded as sections of the clinical notes were missing. Two hundred and three patients were included in the study.

### Patient characteristics

Patient characteristics are shown in Table 1. The mean age of patients was 66.9 years, 65% were male, 55.7% had laparoscopic anterior resection and 44.8% had received neoadjuvant radiotherapy. 69% of patients received an initial defunctioning stoma.

**Table 1:** Patient and tumour characteristics

	*N* (%)
Gender	
Male	132 (65)
Female	71 (35)
Mean age in years (median, range)	66.9 (69, 22–88)
Charlson comorbidity score	
0–3	162 (79.8)
4–5	17 (8.4)
6–7	21 (10.3)
>8	3 (1.5)
ASA score	
1	54 (26.6)
2	92 (45.3)
3	52 (25.6)
4	5 (2.5)
Neoadjuvant therapy	
Receiving radiotherapy in any regimen	91 (44.8)
Long course chemoradiotherapy	78 (38.6)
Radiotherapy only	13 (6.4)
Chemotherapy only	16 (7.9)
Surgical technique	
Laparoscopic	113 (55.7)
Laparoscopic converted to open	13 (6.4)
Open	77 (37.9)
Defunctioning stoma	140 (69)
Loop ileostomy	93 (45.8)
Loop colostomy	47 (23.2)
TNM stage (pathological)	
pCR	11 (5.5)
No residual tumour after local resection	3 (1.5)
*T* stage	
*T*1	13 (6.5)
*T*2	39 (19.5)
*T*3	113 (56.5)
*T*4	21 (10.5)
*N* stage	
*N*0	126 (62.1)
*N*1	51 (25.1)
*N*2	26 (13.8)
*M* stage	
*M*0	183 (90.1)
*M*1	20 (9.9)

Percentages in parenthesis

pCR = complete pathological response to neoadjuvant therapy

Table 2 shows the characteristics of patients with a defunctioning stoma compared to those without. On univariate analysis, there were significant differences between patients who had received neoadjuvant radiotherapy, patients who underwent open surgery, tumour level and the operating surgeon. There were otherwise no significant differences for all other characteristics.

**Table 2:** Univariate analysis comparing characteristics of patients with a defunctioning stoma to those without

Characteristic	Defunctioning stoma		*p* value^a^
	Yes *n* = 140	No *n* = 63	
Male sex	93 (66.4)	39 (61.9)	0.53
ASA score			
1	41 (29.3)	13 (20.6)	
2	66 (47.1)	26 (41.3)	0.18
3	30 (21.4)	22 (34.9)	
4	3 (2.1)	2 (3.1)	
Charlson score			
0–3	110 (78.6)	52 (82.5)	
4–5	12 (8.6)	5 (7.9)	0.89
6–7	16 (11.4)	5 (7.9)	
>8	2 (1.4)	1 (1.6)	
Surgical technique			
Laparoscopic	74 (52.9)	52 (82.5)	<0.001
Open	66 (47.1)	11 (17.5)	
Neoadjuvant radiotherapy	72 (51.4)	19 (30.2)	0.005
Smoker	25 (17.9)	10 (15.9)	0.73
*T*4 tumour	13 (9.3)	8 (12.7)	0.46
Surgeon			
1^b^	39 (27.9)	8 (12.7)	
2	44 (31.4)	8 (12.7)	
3	21 (15)	41 (65)	0.001
4	19 (13.6)	3 (4.8)	
5	17 (12.1)	3 (4.8)	
Mean BMI (kg/m^2^)^c^	26.3	26.7	0.62
Tumour height, mean (mm)^c^	68.0	77.3	0.12
Tumour level			
Upper rectum	41 (29.5)	35 (54.7)	0.003
Mid-rectum	50 (36.0)	16 (25.0)	
Low rectum	48 (34.5)	13 (20.3)	

^a^*Χ*^2^ test analysis unless otherwise stated

^b^Surgeon 1 is the combined operations of surgeons with less than 20 procedures

^c^*T* test

Significant differences identified on univariate analysis were further subjected to multiple logistic regression analysis (Table 3). Only the operating surgeon was independently associated with whether a stoma was formed with one surgeon significantly less likely to use a defunctioning stoma. Surgical technique (open surgery) was not a significant factor.

**Table 3:** Multiple logistic regression analysis of factors associated with creation of a defunctioning stoma

	Odds ratio	95% CI	*p* value
Open surgery	1.74	0.42–7.39	0.45
Neoadjuvant radiotherapy	2.12	0.79–5.66	0.13
Surgeon			
1	1.00 (reference)		
2	1.03	0.23–4.58	0.96
3	0.13	0.05–0.38	<0.001
4	1.51	0.35–6.63	0.58
5	1.72	0.38–7.84	0.48
Level of tumour			
Upper rectal	1.00 (reference)		
Mid-rectal	1.58	0.60–4.21	0.36
Low rectal	2.34	0.75–7.38	0.15

*CI* confidence interval

Table 4 compares patient characteristics according to tumour level. Patients with low rectal tumours were more likely to receive neoadjuvant radiotherapy, in keeping with standard practice in the UK.

**Table 4:** Comparison of patient and treatment characteristics according to tumour level

	Level of rectal cancer			*p* value
	Upper (*n* = 76)	Middle (*n* = 66)	Lower (*n* = 61)	
Age, years^a^	68.6	66.8	65.0	0.20
Charlson score				
≥4	17 (22.4)	12 (18.2)	12 (19.7)	0.82
<4	59 (77.6)	54 (81.8)	49 (80.3)	
BMI (kg/m^2^)^a^	27.5	25.9	25.6	0.08
Neoadjuvant radiotherapy	5 (5.5)	38 (41.8%)	48 (52.8)	<0.001
Surgical technique				
Open	29 (38.2)	30 (45.5)	18 (29.5)	0.18
Laparoscopic	47 (61.9)	36 (54.6)	43 (70.5)	

Percentages in parenthesis unless otherwise stated

Comparisons using *Χ*^2^ test unless otherwise stated

*OR* odds ratio, CI confidence interval

^a^*T* test, means

### Outcomes

The overall 90-day complication rate was 50.2% and 2 (1%) patients died within 90 days of surgery.

Patients who had a defunctioning stoma created had significantly more overall complications than those managed without a stoma (57.1 vs 34.9%, OR 2.48, *p* = 0.003)(Table 5).

**Table 5:** Comparison of 90-day post-operative outcomes between patients managed with and without defunctioning stomas

Outcome	Defunctioning stoma *n* (%)		OR (95% CI)	*p* value
	Yes	No		
Overall complications	80 (57.1)	22 (34.9)	2.48 (1.34–4.60)	0.003
Anastomotic leak	19 (13.6)	9 (14.3)	0.94 (0.40–2.22)	0.89
Abdominal sepsis	26 (18.6)	13 (20.7)	0.87 (0.42–1.85)	0.73
Ileus	18 (12.9)	1 (1.6)	9.37 (1.22–71.8)	0.009
Bowel obstruction	9 (6.4)	0 (0)	Undefined	0.06^a^
Complications according to severity				
Clavien–Dindo 1	14 (10.0)	1 (1.6)		<0.001
Clavien–Dindo 2	45 (32.1)	7 (11.1)		
Clavien–Dindo 3a	5 (3.6)	0 (0)		
Clavien–Dindo 3b	9 (6.4)	11 (17.5)		
Clavien–Dindo 4a	4 (2.9)	2 (3.2)		
Clavien–Dindo 4b	2 (1.4)	0 (0)		
Clavien–Dindo 5	1 (0.7)	1 (1.6)		
Significant complications (Clavien–Dindo ≥ 2)	66 (47.1)	21 (33.1)	1.78 (0.96–3.32)	0.06
Multiple complications	25 (17.9)	2 (3.2)	6.63 (1.52–28.9)	0.004
Reoperation	11 (7.9)	13 (20.6)	0.33 (0.14–0.78)	0.009
Readmission	27 (19.6)	14 (22.2)	0.85 (0.41–1.76)	0.66
Mortality	1 (0.71)	1 (1.59)	0.45 (0.03–7.25)	0.56
Length of hospital stay in days^a^	13.0 (8)	6.9 (4)		0.005
Total length of hospital stay in days^a^	14.1 (9)	10.7 (5)		0.15

Percentages in parenthesis unless otherwise stated

Comparisons using *Χ*^2^ test unless otherwise stated

Total length of hospital stay includes duration of stay for unplanned readmissions but not hospital stay associated with stoma reversal or subsequent complications

*OR* odds ratio, CI confidence interval

^a^*T* test, means

Patients managed with a defunctioning stoma were less likely to require reoperation. There were no significant differences between groups in anastomotic leak rates or abdominal sepsis overall (anastomotic leaks, enterotomy or intraabdominal collection not meeting the definition of anastomotic leak), readmissions or mortality. However, patients who received a primary stoma were significantly more likely to develop post-operative ileus and to experience multiple complications.

Patients managed with a defunctioning stoma had a mean primary length of hospital stay of 13 days compared to 6.9 days (*p* = 0.005).

Table 6 shows univariate analysis of potential patient and surgical factors associated with post-operative complications, and those with *p* value <0.2 were subjected to multiple logistic regression to identify independent associations (Table 7). Only the presence of a defunctioning stoma and ASA score ≥3 were independently associated with post-operative complications.

**Table 6:** Univariate analysis of patient and surgical factors and post-operative complications

	Post-operative complication, *n* (%)	OR (95% CI)	*p* value
Defunctioning stoma			
Yes	80 (57.1)	2.48 (1.34–4.60)	0.003
No	22 (34.9)		
Surgical technique			
Open	38 (49.4)	0.94 (0.54–1.66)	0.84
Laparoscopic	64 (50.8)		
Surgeon			
1	24 (51.1)		0.05
2	23 (44.2)		
3	26 (41.9)		
4	17 (77.3)		
5	12 (60.0)		
Level of tumour			
Upper rectal	31 (40.8)		0.11
Mid-rectal	37 (56.1)		
Low rectal	34 (55.7)		
Neoadjuvant radiotherapy			
Yes	46 (50.6)	1.02 (0.59–1.78)	0.94
No	56 (50.0)		
Operation time (mins)			
<180	36 (47.4)		0.01
180–239	17 (37.8)		
240–299	11 (42.3)		
≥300	38 (67.9)		
ASA score ≥ 3			
Yes	34 (59.7)	1.70 (0.91–3.16)	0.09
No	68 (46.6)		
Charlson score ≥ 4			
Yes	24 (58.5)	1.52 (0.76–3.04)	0.23
No	78 (48.2)		
Smoking			
Yes	18 (51.4)	1.06 (0.51–2.19)	0.88
No	84 (50.0)		
Age			
>70 years	42 (48.9)	0.91 (0.52–1.58)	0.73
<70 years	60 (51.3)		
BMI			
≥30 kg/m^2^	11 (45.8)	0.86 (0.36–2.1)	0.75
<30 kg/m^2^	50 (49.5)		

Percentages in parenthesis

*OR* odds ratio, *CI* confidence interval

**Table 7:** Multiple logistic regression analysis of factors associated with post-operative complications

	Odds ratio	95% CI	*p* value
Defunctioning stoma	2.62	1.19–5.78	0.02
Surgeon			
1	1.00 (reference)		
2	0.73	0.24–2.24	0.58
3	1.30	0.47–3.57	0.61
4	2.53	0.75–8.56	0.14
5	0.99	0.30–3.19	0.98
Level of tumour			
Upper rectal	1.00 (reference)		
Mid-rectal	1.85	0.86–3.98	0.12
Low rectal	1.53	0.70–3.33	0.29
ASA ≥ 3	2.39	1.15–4.98	0.02
Operation time (min)			
<180	1.00 (reference)		
180–239	0.60	0.25–1.44	0.25
240–299	0.60	0.18–1.92	0.38
≥300	1.46	0.42–5.01	0.55

### Patients requiring reoperation

Eleven patients with a defunctioning stoma required a reoperation as a result of complications, which included 7 patients requiring an unplanned abdominal reoperation as follows: 2 patients required an urgent early reversal of stoma, 2 patients required laparotomy and revision of their stoma for ischaemic necrosis, 2 patients required laparotomy for lavage (one for an intraabdominal collection and another for an anastomotic leak) and one required laparotomy and adhesiolysis to relieve small bowel obstruction. The other 4 patients with defunctioning stomas requiring reoperations all received examination under anaesthesia, lavage ± transrectal drainage for anastomotic leak (*n* = 3) or suspected haemorrhage from the anastomosis (*n* = 1). By contrast, 11 patients who did not receive an initial defunctioning stoma required an abdominal operation as a result of post-operative complications. Most abdominal operations in this group were to treat intraabdominal sepsis secondary to anastomotic leak, with 9 patients having a stoma formed at reoperation. Only one patient required the anastomosis to be taken down and an end colostomy formed. Five of the other 8 patients who subsequently received a stoma had the reoperation performed laparoscopically without the need to resort to laparotomy.

### Stoma-related complications

Thirty-seven stoma-related complications occurred in 27 (19.3%) patients within 90 days. Four patients required urgent or unplanned reoperations as a result of stoma-related complications. The most common stoma-related complication was high output (*n* = 18), which resulted in acute kidney injury in 6 patients. Three patients had bowel obstruction which on radiological studies appeared directly related to the stoma, and 6 others in this group developed bowel obstruction at a site other than the stoma (distinguished from ileus by radiological features on CT scan).

Seventy-eight patients (55.7%) who initially received a defunctioning stoma still had the stoma 1 year later. There were many and varied reasons for this including anastomotic stricture requiring treatment (*n* = 11), medically unwell or too frail for further surgery (*n* = 11), post-operative complications after original surgery (*n* = 10), local or distant recurrence detected (*n* = 14), complications with adjuvant chemotherapy delaying stoma closure (*n* = 3), suspected abnormalities detected on radiological imaging requiring further investigations (*n* = 4), other miscellaneous (*n* = 5) and several due to long waiting list times (*n* = 20). Thirty-six patients (25.7%) remained with a stoma at the time of last follow-up (mean follow-up 34.4 months). Six (9.5%) patients who did not initially receive a defunctioning stoma remained with a stoma at last follow-up.

## Discussion

Several studies, including multiple meta-analyses, suggest that the use of a defunctioning stoma in anterior resection reduces the risk or diminishes the consequences of an anastomotic leak [4–7, 18, 19, 25–30]. However, the magnitude of this benefit is uncertain and there are no accurate means of identifying which patients will benefit from a defunctioning stoma. It is clear that, in contemporary practice, the majority of patients undergoing anterior resection for rectal cancer receive an initial defunctioning stoma, making the practice almost routine in many centres [1–3]. Despite these high rates of defunctioning stoma use, there are very few studies comparing overall complication rates in patients who are managed with a defunctioning stoma to those without [8–10], and few of the RCTs or non-randomised studies examining the effect of defunctioning stomas on anastomotic leak report overall complications [18, 27, 29]. Evidence of the effect of forming a defunctioning stoma on overall short-term morbidity, which is currently lacking, may well influence a surgeon’s decision to create a stoma during anterior resection.

We found that patients who have a defunctioning stoma created during anterior resection have an increase in overall complications of 22%. Patients with a defunctioning stoma were more likely to suffer multiple complications, almost 20% experienced ileus or bowel obstruction and almost 20% experienced a stoma-related complication. The rates of anastomotic leak or abdominal sepsis in general were not significantly different in patients with and without a defunctioning stoma.

These results contrast with the only other published study including over 200 patients comparing complication rates in which Anderin et al. [8] reported a complication rate among patients with a defunctioning stoma of 53% compared to 43% in those without. However, this study reported 30-day post-operative complications. A significant number of adverse post-operative outcomes are missed when only considering 30-day outcomes compared to 90-day outcomes [31, 32] and it is possible that this partly accounts for the higher morbidity found in our study. It is also noteworthy that only 15% of patients received a defunctioning stoma in the initial period of that study compared to 91% during the latter period, and the rate of anastomotic leak did not differ between these two periods leading the authors to call into question such routine use of stomas.

In a smaller study, Ihnát et al. [33] found significantly higher overall morbidity in patients managed with a defunctioning stoma compared to those without (42.3 vs 23.3%), although only laparoscopic anterior resection was considered. Gumbau et al. compared outcomes in a small group of patients and found no significant differences in overall complications, however this study may have lacked a sufficient sample size and appears to have included all anterior resections rather than selecting low anterior resections. Only 4 patients who had TME did not receive a defunctioning stoma.

The magnitude of any benefit of defunctioning stomas is hugely variable among studies, reflected in a systematic review and meta-analysis in which the pooled rate of anastomotic leak and reoperation in low anterior resection showed a benefit in favour of defunctioning stomas of less than 2% [34]. Given the possibility that a defunctioning stoma may only offer a relatively small absolute benefit, an increase in complications of 22% in patients with a defunctioning stoma should prompt surgeons to carefully consider the near routine use of stomas in anterior resection. Even the most generous interpretation of the literature would conclude that the majority of patients receiving defunctioning stomas will not derive any benefit, and therefore subjecting 80% of patients undergoing anterior resection to a defunctioning stoma, as is the case in many centres [1–3, 33], means subjecting many patients to this significantly increased risk of complications.

It is important to consider the consequences of more selective use of defunctioning stomas. Nine patients who did not have an initial defunctioning stoma went on to have a stoma created at a subsequent reoperation for a suspected anastomotic leak. One had a further resection and formation of an end colostomy as a result of extensive ischaemic necrosis. Two patients underwent laparotomy and formation of a defunctioning stoma. The remaining 6 patients had a laparoscopic procedure; in 2 patients no anastomotic leak was discovered but a defunctioning stoma was formed in any case. The other 4 patients had laparoscopic lavage and formation of a defunctioning stoma. Four patients had the stoma closed. Therefore 14% of patients who did not initially receive a defunctioning stoma subsequently had a stoma formed at reoperation and 9.5% remain with what could be considered a permanent stoma compared to 26% of patients who received an initial “temporary” defunctioning stoma. This demonstrates that selective use of defunctioning stomas results in far fewer patients ultimately requiring a stoma, as well as far fewer patients remaining with what could be considered a permanent stoma after a mean follow-up of almost 3 years. This figure may initially seem surprising, but it reflects common practice and is similar to other published findings, including national figures [35, 36]. We feel it is a very important and often overlooked consideration in deciding to form a defunctioning stoma, and one about which patients are very seldom informed.

Although a plethora of potential risk factors for anastomotic leak have been identified [37], none have definitively emerged to help surgeons decide which patients would benefit from a defunctioning stoma, leaving surgeons basing the decision on preconceived ideas of significant risk factors. For example, many surgeons consider neoadjuvant chemoradiotherapy to be a significant risk factor for anastomotic leak; however, there is strong evidence that this is not the case [38, 39]. Also, the decision to create a stoma could be related to a surgeon’s age or personality with one study showing that younger surgeons and those with a lower propensity for risk-taking were more likely to decide to create a defunctioning stoma [40]. Routinely creating defunctioning stomas based on unproven risk factors for anastomotic leak or a surgeon’s personality traits might lead to patients, many elderly with significant comorbidities, being subjected to a significant increased risk of complications or remaining with a permanent stoma. Forming a stoma is a significant additional procedure which unsurprisingly could result in additional complications although some findings in this study, such as the much higher risk of developing ileus after stoma formation, are somewhat less self-evident. We believe the additional manipulation and handling of the small bowel required to form a stoma could lead to ileus, especially during a laparoscopic procedure during which very minimal manipulation of the small bowel is performed.

It is also important to address concerns about higher reoperation rates in patients who do not receive a defunctioning stoma. Although a higher portion of these patients undergoes reoperations, this study shows that in many cases these can be completed laparoscopically thus minimising the impact.

### Limitations

There are several limitations to the current study. This was not a randomised controlled study and is therefore subject to selection bias. It is likely that many patients with a defunctioning stoma were considered to be at higher risk of anastomotic leak by the operating surgeon and that the results of this study reflect good patient selection by the operating surgeon, particularly by those surgeons who tend to fashion fewer defunctioning stomas.

A higher proportion of patients receiving a defunctioning stoma in this study had been treated with neoadjuvant chemotherapy or radiotherapy but the groups were well matched in all other respects. It is possible that other risk factors which were not recorded in this study influenced the surgeon’s decision to fashion a defunctioning stoma and these may have predisposed patients to developing post-operative complications. However, there were no differences between groups in mean age, sex, smoking status, BMI, T4 tumours, ASA or Charlson comorbidity scores and it is therefore difficult to speculate on what other risk factors for complications may have been present. We also included as homogeneous a patient population as possible by using objective methods in deciding which patients had a low anterior resection and should be included in the study. The definition varies widely among studies on defunctioning stomas and the majority do not specify the method of measurement. We feel that strategies such as assessment on digital rectal examination or colonoscopy are inaccurate in assessing the tumour level, and rigid sigmoidoscopy was not consistently performed on every patient in this study and is usually done with an unprepared rectum in which tumours were sometimes missed, and was therefore neither as widely available nor objectively reproducible as MRI or the histopathological analysis for this study.

A higher proportion of patients who had laparoscopic surgery were managed without a defunctioning stoma compared to those who had open surgery. However, open surgery was eliminated as a significant factor associated with the decision to create a defunctioning stoma on multiple regression analysis. The difference in the proportion of patients who received a defunctioning stoma was more likely driven by the preference for a more selective use of defunctioning stomas by one of the laparoscopic surgeons compared to those practising mostly open surgery. On multivariate analysis, the operating surgeon, tumour level, length of operation and neoadjuvant radiotherapy were not independently associated with complications. Only the presence of a defunctioning stoma and ASA score ≥3, and not open surgery, were associated with a significantly increased post-operative complication rate, in keeping with evidence that open surgery is not associated with more complications than laparoscopic surgery in rectal resection, with 2 recent well-conducted randomised controlled trials in rectal cancer resection showing complication rates for open and laparoscopic surgery [41, 42].

45% of patients in this study received neoadjuvant radiotherapy, which may be lower than similar cohorts in the USA but is, however, very similar to rates of neoadjuvant radiotherapy to rectal cancer patients in the UK, much of Europe as well as Australia and is therefore representative of other rectal cancer patient cohorts undergoing surgery [1, 41, 43].

## Conclusion

This study shows that patients selected to have a defunctioning stoma at anterior resection have a significantly increased risk of overall complications, are more likely to suffer multiple complications and have a longer primary hospital stay. Almost 1 in 5 patients suffered a stoma-related complication and 26% remained with a stoma at last follow-up compared to 9.5% of other patients. These findings support the more selective use of stomas in anterior resection.
